# Inositol and Berberine Synergistically Reprogram Endocrine and Ovarian Metabolism in Polycystic Ovary Syndrome

**DOI:** 10.1111/cpr.70188

**Published:** 2026-02-27

**Authors:** Juan Ge, Chenxiang Zhang, Yilong Zhao, Haiyang Zhao, Shuai Zhu, Tao Tang, Guorui Zhang, Qiang Wang, Hui Wang

**Affiliations:** ^1^ State Key Laboratory of Reproductive Medicine and Offspring Health, Changzhou Maternity and Child Health Care Hospital, Changzhou Medical Center Nanjing Medical University Nanjing China; ^2^ Nanjing BioDe Biotechnology co., Ltd. Nanjing China; ^3^ Chinese Medicine Research Institute, Engineering Research Center of Modern Preparation Technology of Traditional Chinese Medicine, Ministry of Education Shanghai University of Traditional Chinese Medicine Shanghai China; ^4^ Changzhou Maternity and Child Health Care Hospital, Changzhou Medical Center, State Key Laboratory of Reproductive Medicine and Offspring Health Nanjing Medical University Nanjing China; ^5^ State Key Laboratory of Reproductive Medicine and Offspring Health, Department of Histology and Embryology, School of Basic Medical Sciences Nanjing Medical University Nanjing China

**Keywords:** berberine, granulosa cell, inositol, polycystic ovary syndrome, reproduction

## Abstract

Although Myo‐Inositol/D‐Chiro‐Inositol (Ins) and berberine (BBR) have each shown beneficial effects in polycystic ovary syndrome (PCOS), their combined therapeutic potential has not been systematically evaluated. Here, we demonstrate that Ins/BBR exerts superior efficacy compared with single treatments by targeting multiple pathogenic pathways in PCOS. In a DHEA+HFD‐induced mouse model, Ins/BBR restored systemic sex steroid balance, normalized LH/FSH ratio, and improved estrous cyclicity. It also reduced ovarian cysts and enhanced fertility, accompanied by partial normalization of steroidogenic enzyme expression. At the cellular level, Ins/BBR alleviated mitochondrial defects and broadly reprogrammed metabolic landscape in granulosa cells, in specific, restoring nucleotide pools and amino acid turnover and preventing abnormal long‐chain fatty acid accumulation. Together, these findings provide preclinical evidence that Ins/BBR acts through coordinated endocrine, ovarian and metabolic mechanisms, supporting its promise as a safe and effective therapeutic strategy for PCOS.

## Graphical abstract

In a DHEA+HFD‐induced mouse model, Ins/BBR alleviated mitochondrial defects and broadly reprogrammed metabolic landscape in granulosa cells, in specific, restoring nucleotide pools and amino acid turnover, and preventing abnormal long‐chain fatty acid accumulation.

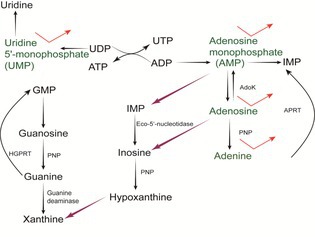

## 1 Introduction

Polycystic Ovary Syndrome (PCOS) is a common endocrine and metabolic disorder, affecting 6%–20% of women of reproductive age worldwide. It is a leading cause of infertility, hyperandrogenism, menstrual irregularities and metabolic disturbances such as insulin resistance and dyslipidemia [1, 2, 3]. PCOS also has long‐term health implications, including increased risks of type 2 diabetes, cardiovascular disease, and endometrial cancer [4, 5, 6]. PCOS is diagnosed using the Rotterdam 2003 criteria, which require at least two of the following three features: oligo‐ovulation/anovulation, hyperandrogenism and polycystic ovaries [7, 8, 9]. Despite extensive research, the exact pathophysiology of PCOS remains unclear. Insulin resistance, affecting up to 85% of women with PCOS, is thought to play a central role. This resistance is often linked to defects in the inositolphosphoglycans (IPGs) second messenger pathway, which is essential for glucose metabolism [5, 10, 11].

Inositol therapies, specifically Myo‐Inositol (MyoIns) and D‐Chiro‐Inositol (DClns), have gained considerable attention for their role in restoring balance in the IPG pathway. MyoIns regulates glucose uptake and follicle‐stimulating hormone signalling, while DClns is involved in glycogen synthesis and androgen production [12, 13]. When combined at a physiological ratio of 40:1, MyoIns/DClns have shown significant promise in treating the metabolic and hormonal abnormalities associated with PCOS [13, 14, 15]. Berberine (BBR), a natural isoquinoline alkaloid found in plants such as Coptis Chinensis, has demonstrated anti‐inflammatory, antioxidant and insulin‐sensitizing effects. It activates AMP‐activated protein kinase (AMPK) and regulates the insulin receptor substrate‐1 pathway, improving insulin sensitivity and metabolic function [16]. BBR has also shown efficacy in improving ovarian function, increasing ovulation rates and enhancing oocyte/embryo quality in PCOS patients [17].

Although MyoIns, DClns and BBR have shown promising individual effects in managing PCOS, the combined therapeutic potential of MyoIns/DClns with BBR remains unexplored. This study aims to compare the effects of MyoIns/DClns, BBR and their combination on the endocrine, metabolic and ovarian phenotypes in a PCOS mouse model. Our findings reveal that the combination of MyoIns/DClns with BBR provides a more effective treatment strategy for alleviating PCOS symptoms.

## 2 Results

### 2.1 Berberine Exerts Dose‐Dependent Improvements in Endocrine and Ovarian Phenotypes of PCOS Mice

Given the established role of berberine (BBR) in reducing plasma triglycerides and improving insulin sensitivity [16, 18], and considering that insulin resistance is central to the pathophysiology of PCOS [19, 20], we sought to determine the optimal BBR concentration for correcting endocrine disturbances and ovarian morphology in a PCOS mouse model.

**FIGURE 1:**
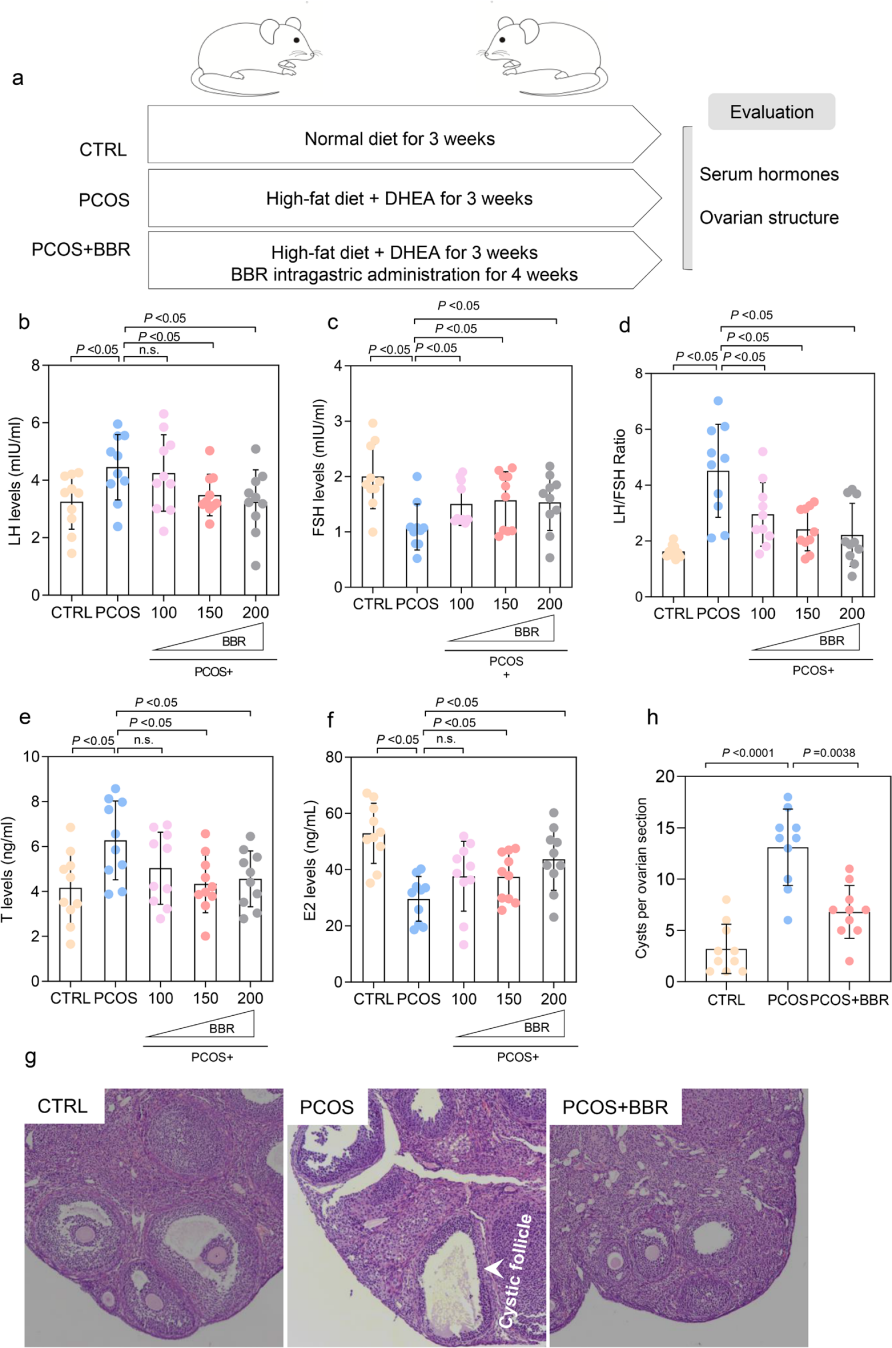
Effects of berberine (BBR) at different concentrations on serum hormone levels and ovarian morphology in PCOS mice. (a) Schematic diagram of PCOS induction and BBR intervention. (b–f) Serum hormone levels of LH, FSH, testosterone (T) and estradiol (E2), as well as the LH/FSH ratio, in CTRL, PCOS and PCOS mice treated with different BBR concentrations (100, 150 or 200 mg/kg/day). (g) Representative ovarian histology showing normal follicular structures in CTRL mice, multiple cystic follicles (white arrows) in PCOS mice, and reduced cystic follicles in PCOS mice treated with 150 mg/kg/day BBR. (h) Quantification of cystic follicle numbers across groups. Scale bar = 200 μm. Data are presented as mean ± SEM (*n* = 3).

To establish the model, three‐week‐old female B6 mice were fed a high‐fat diet and given daily subcutaneous injections of dehydroepiandrosterone (DHEA) for 21 consecutive days, an approach widely recognized to mimic both the reproductive and metabolic phenotypes of PCOS. PCOS induction resulted in elevated serum testosterone (TP) and luteinizing hormone (LH) levels, along with reduced follicle‐stimulating hormone (FSH) and estradiol (E2). Intragastric administration of BBR at three concentrations (100, 150 and 200 mg/kg/day) for 28 days demonstrated that 150 mg/kg/day produced the most pronounced therapeutic effects. Specifically, mice in the PCOS+BBR/150 group showed a marked decline in TP, LH and LH/FSH ratio, together with significant restoration of FSH and E2 compared with control groups (Figure 1b–f). Histological examination revealed typical cystic follicle formation in untreated PCOS ovaries, whereas BBR treatment substantially reduced the number of cystic follicles, with the most notable improvement again observed in the 150 mg/kg/day group (Figure 1g–h). These findings indicate that BBR exerts a dose‐dependent effect in ameliorating endocrine abnormalities and ovarian cystic morphology in PCOS mice, with 150 mg/kg/day identified as the optimal dose for subsequent experiments.

### 2.2 Ins/BBR Combination Restores Hormonal Balance in PCOS Mice

**FIGURE 2:**
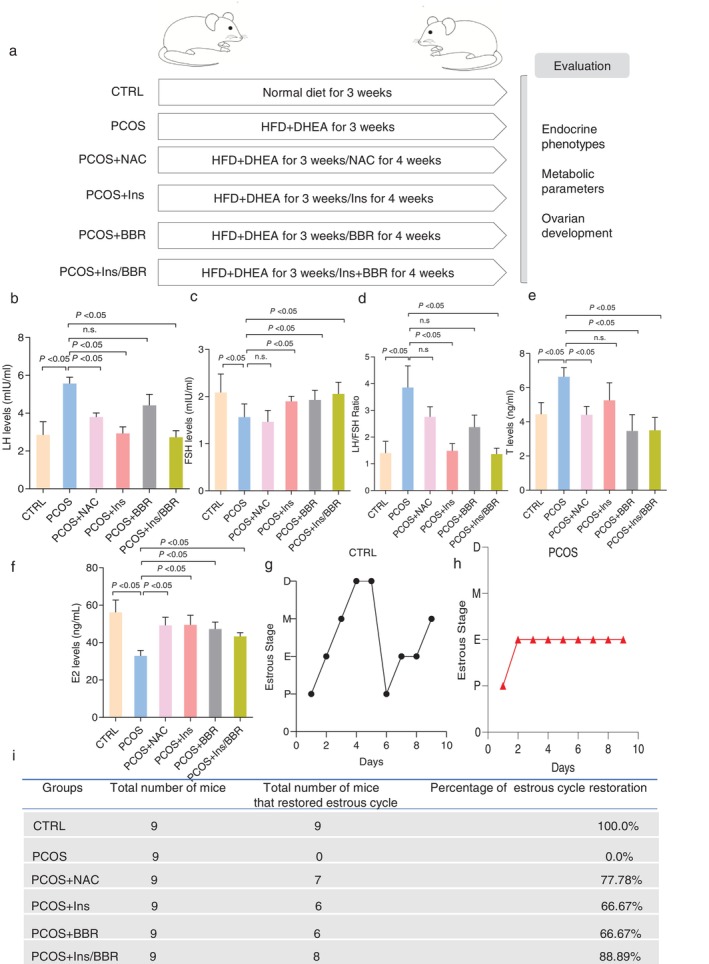
Endocrine phenotypes and estrous cycle restoration in PCOS mice following different interventions. (a) Experimental protocol for PCOS induction and treatment with NAC, Ins, BBR or Ins/BBR. (b–f) Serum hormone levels of LH, FSH, testosterone (T) and estradiol (E2), as well as the LH/FSH ratio, across groups. (g–h) Representative estrous cycle monitoring over 10 consecutive days in CTRL and PCOS mice. (i) Percentage of mice with restored estrous cycles in each group. Data are presented as mean ± SD from ≥ 3 independent experiments.

Myo‐Inositol/D‐Chiro‐Inositol (Ins) and berberine (BBR) have been shown to prevent endocrine and reproductive abnormalities in PCOS [21, 22]. N‐acetyl‐cysteine (NAC), a precursor of L‐cysteine, also protects against oxidative stress and ovarian metabolic dysfunction in PCOS models [23, 24, 25]. Here, we compared the effects of NAC, Ins, BBR and their combination (Ins/BBR) on phenotypic defects in a PCOS mouse model (Figure 2a).

To do this, HFD + DHEA‐induced PCOS mice were administrated respectively using gavage needles with NAC (300 mg/kg/day), Ins (40:1; 310 mg/kg/day), BBR (150 mg/kg/day), and Ins/BBR (40:1:18) for 28 consecutive days. Remarkably, compared with single treatments, Ins/BBR more effectively corrected hormonal imbalances in PCOS mice, leading to reduced LH/FSH ratio and normalized TP and E2 levels (Figure 2b–f). Meanwhile, estrous cycle monitoring over ten consecutive days revealed that untreated PCOS mice were arrested in prolonged estrus. While NAC, Ins and BBR each partially restored cyclicity. In contrast, Ins/BBR treatment showed the most pronounced improvement, re‐establishing regular cycles with sequential proestrus, estrus, metestrus and diestrus phases (Figure 2g–i). Together, Ins/BBR synergistically restored endocrine balance and normalized estrous cycles in PCOS mice, demonstrating superior efficacy over single‐agent interventions in improving reproductive function.

### 2.3 Ins/BBR Combination Synergistically Alleviates Metabolic Dysfunction in PCOS Mice

Insulin resistance and obesity are central metabolic features of PCOS. To evaluate whether Ins, BBR, NAC or their combination (Ins/BBR) could improve these metabolic abnormalities, we monitored body weight, fat mass and glucose homeostasis in PCOS mice.

**FIGURE 3:**
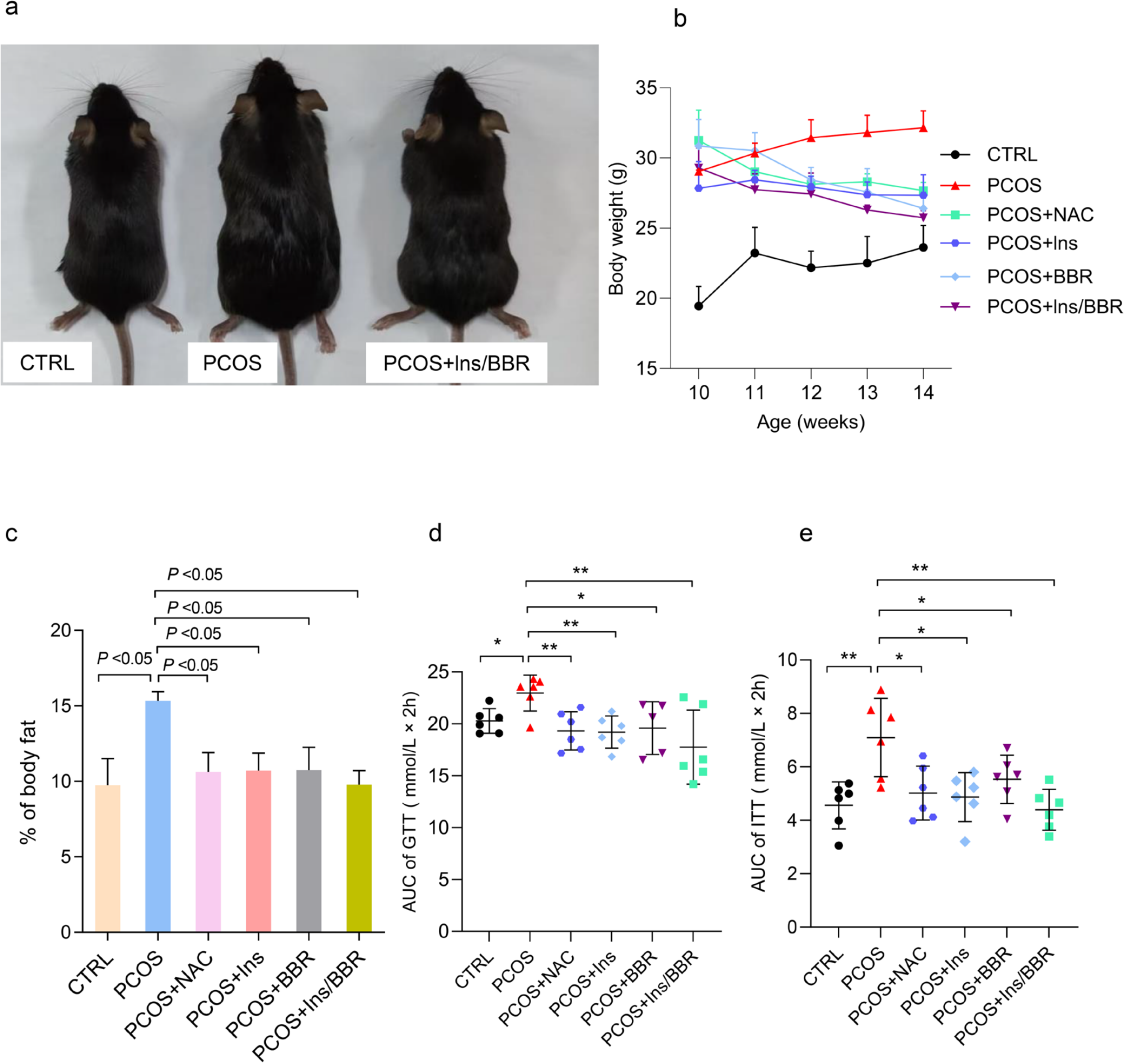
Effects of Ins, BBR, NAC and Ins/BBR on body weight and glucose homeostasis in PCOS mice. (a) Representative body appearance of CTRL, PCOS and treatment groups. (b) Body weight changes over four weeks of intervention. (c) Percentage of fat mass in different groups. (d) Glucose tolerance test (GTT) and area under the curve (AUC) in CTRL, PCOS, and treated groups following glucose injection (2 g/kg for CTRL, 1 g/kg for others; *n* = 5–6 per group). (e) Insulin tolerance test (ITT) and AUC after insulin injection (0.01 mL/g), with blood glucose monitored for 120 min. Data are presented as mean ± SEM. *p < 0.05, **p < 0.01.

PCOS mice showed a significant increase in body weight compared with controls (Figure 3a). All interventions reduced body weight, with Ins/BBR producing the most pronounced effect (Figure 3b). A similar trend was observed in fat mass percentage (Figure 3c). Glucose tolerance tests (GTT) and insulin tolerance tests (ITT), assessed by area under the curve (AUC), and revealed severe glucose intolerance and insulin resistance in untreated PCOS mice. After 28 days of intervention, fasting blood glucose and insulin levels were significantly decreased in the NAC, Ins, BBR and Ins/BBR groups, nearly reaching control levels. Among these, Ins/BBR treatment exhibited the most substantial improvement in both glucose tolerance and insulin sensitivity (Figure 3d–e). These observations suggest that Ins/BBR effectively alleviated obesity, glucose intolerance and insulin resistance in PCOS mice, demonstrating synergistic benefits on systemic metabolic regulation.

### 2.4 Ins/BBR Combination Prevents Ovarian Abnormalities in PCOS Mice

Ovulatory dysfunction in PCOS is the leading cause of anovulatory infertility and is associated with pathological changes in germ cells, granulosa cells, and theca cells [26, 27]. Ovarian reserve, defined by follicle number and oocyte quality, is a key marker of female fertility [28, 29]. Hence, we assessed whether Ins, BBR, NAC or their combination (Ins/BBR) could restore ovarian morphology and folliculogenesis in PCOS mice.

**FIGURE 4:**
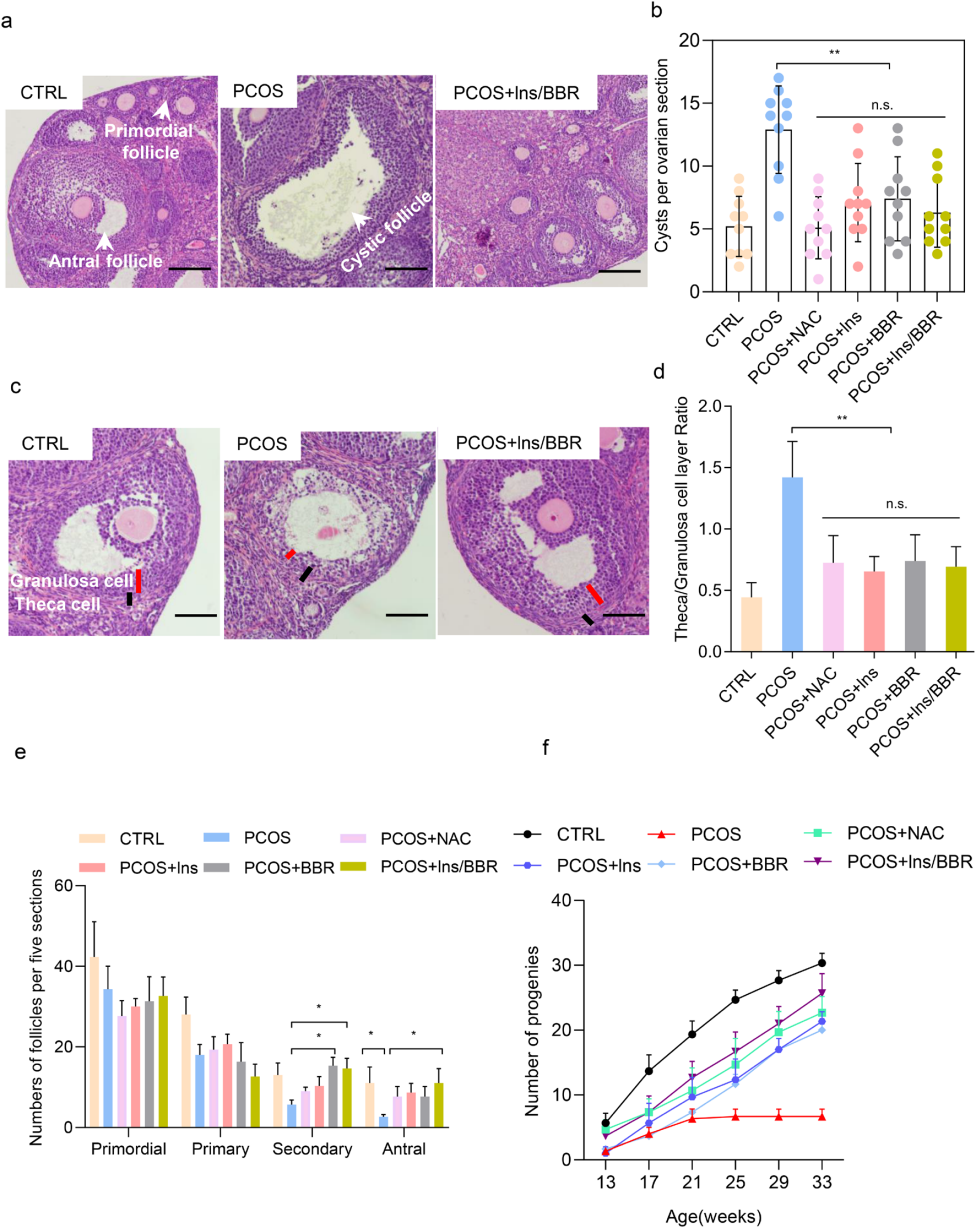
Ovarian morphology and fertility outcomes in PCOS mice treated with Ins, BBR, NAC or Ins/BBR. (a) Representative ovarian sections showing different follicle types in CTRL, PCOS and PCOS+Ins/BBR groups. (b) Quantification of cystic follicles per ovarian section across groups (scale bar = 200 μm). (c) Representative images of theca cell layer (T, black lines) and granulosa cell layer (G, red lines) thickness. (d) Ratio of theca/granulosa cell layer thickness across groups. (e) Quantification of follicle types per section in different groups. (f) Cumulative progeny number during 20 weeks of mating across groups. Data are mean ± SEM (*n* = 4). *p < 0.05, **p < 0.01; n.s., not significant. Scale bar = 20 μm.

H&E staining revealed normal follicular architecture (primordial, primary, secondary and antral follicles) in control and Ins/BBR‐treated ovaries, whereas untreated PCOS ovaries exhibited multiple cystic follicles (Figure 4a–b). Ins/BBR treatment significantly reduced cyst formation and preserved follicular structures. In PCOS ovaries, theca cell layers were thickened and granulosa layers reduced, disturbing the physiological TC/GC ratio. All pharmacological interventions partially normalized these histological abnormalities (Figure 4c–d). Quantitative analysis revealed no significant difference in primordial or primary follicle numbers between groups, consistent with previous studies [30]. However, secondary and antral follicles were markedly reduced in PCOS mice compared with controls, and this decline was effectively prevented by Ins/BBR treatment (Figure 4e). Furthermore, continuous breeding assays further confirmed impaired fertility in PCOS mice, which exhibited reduced cumulative litter size over 20 weeks. Ins/BBR administration significantly enhanced reproductive capacity compared with untreated PCOS mice (Figure 4f).

Altogether, Ins/BBR restored ovarian morphology by reducing cystic follicles, normalizing GC/TC structure, and preserving secondary/antral follicle populations. These improvements translated into enhanced fertility, underscoring the therapeutic potential of Ins/BBR in correcting reproductive dysfunction in PCOS.

### 2.5 Ins/BBR Combination Improves Mitochondrial Function in Granulosa Cells From PCOS Mice

Granulosa cells are critical for oocyte maturation, steroid hormone production, and follicular development. To explore whether Ins, BBR, NAC or their combination (Ins/BBR) could restore granulosa cell function in PCOS, we assessed mitochondrial structure, biogenesis, energy production and steroidogenic gene expression.

**FIGURE 5:**
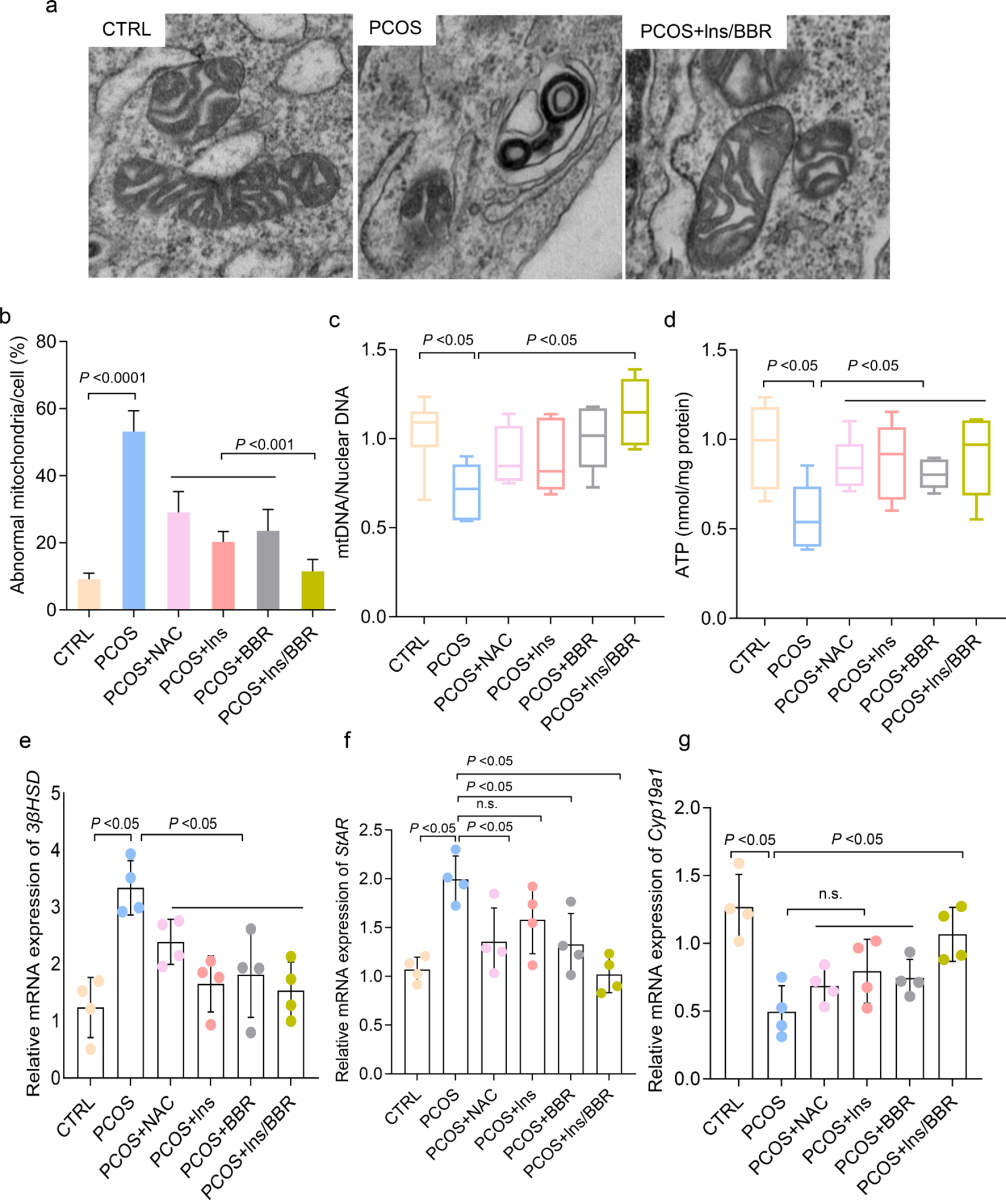
Mitochondrial morphology and steroidogenic gene expression in granulosa cells from PCOS mice. (a) Transmission electron microscopy (TEM) images of granulosa cell mitochondria in CTRL, PCOS and PCOS+Ins/BBR groups. (b) Percentage of abnormal mitochondria per cell. (c) mtDNA copy number determined by RT‐qPCR. (d) Intracellular ATP content in granulosa cells across groups. Data are mean ± SD from three independent experiments. (e–g) Relative mRNA expression of 3βHSD, StAR and Cyp19a1 in granulosa cells from CTRL, PCOS and treated groups. *p < 0.05; n.s., not significant.

Electron microscopy revealed severe mitochondrial abnormalities in granulosa cells from PCOS mice, characterized by disrupted or absent cristae (Figure 5a). Ins/BBR treatment markedly reduced the proportion of damaged mitochondria (Figure 5b). Consistently, mtDNA copy number was significantly decreased in PCOS granulosa cells but restored after pharmacological interventions, with Ins/BBR showing the greatest improvement (Figure 5c). ATP levels were also significantly reduced in PCOS granulosa cells (0.56 nmol/mg), whereas all treatment groups displayed higher levels (> 0.81 nmol/mg), indicating recovery of mitochondrial bioenergetics (Figure 5d). Steroidogenic gene analysis revealed dysregulated expression in PCOS granulosa cells: *StAR* and *3βHSD* were upregulated, while *Cyp19a1* (aromatase) was reduced. Pharmacological treatments generally normalized *StAR* and *3βHSD* expression, with Ins/BBR exerting the strongest effect. However, *Cyp19a1* expression showed no significant recovery in the NAC, Ins or BBR groups alone (Figure 5e–g). These findings indicate that Ins/BBR effectively corrected mitochondrial status and partially normalized steroidogenesis in PCOS granulosa cells.

### 2.6 Ins/BBR Combination Broadly Reprograms Granulosa Cells Metabolism in PCOS Mice

**FIGURE 6:**
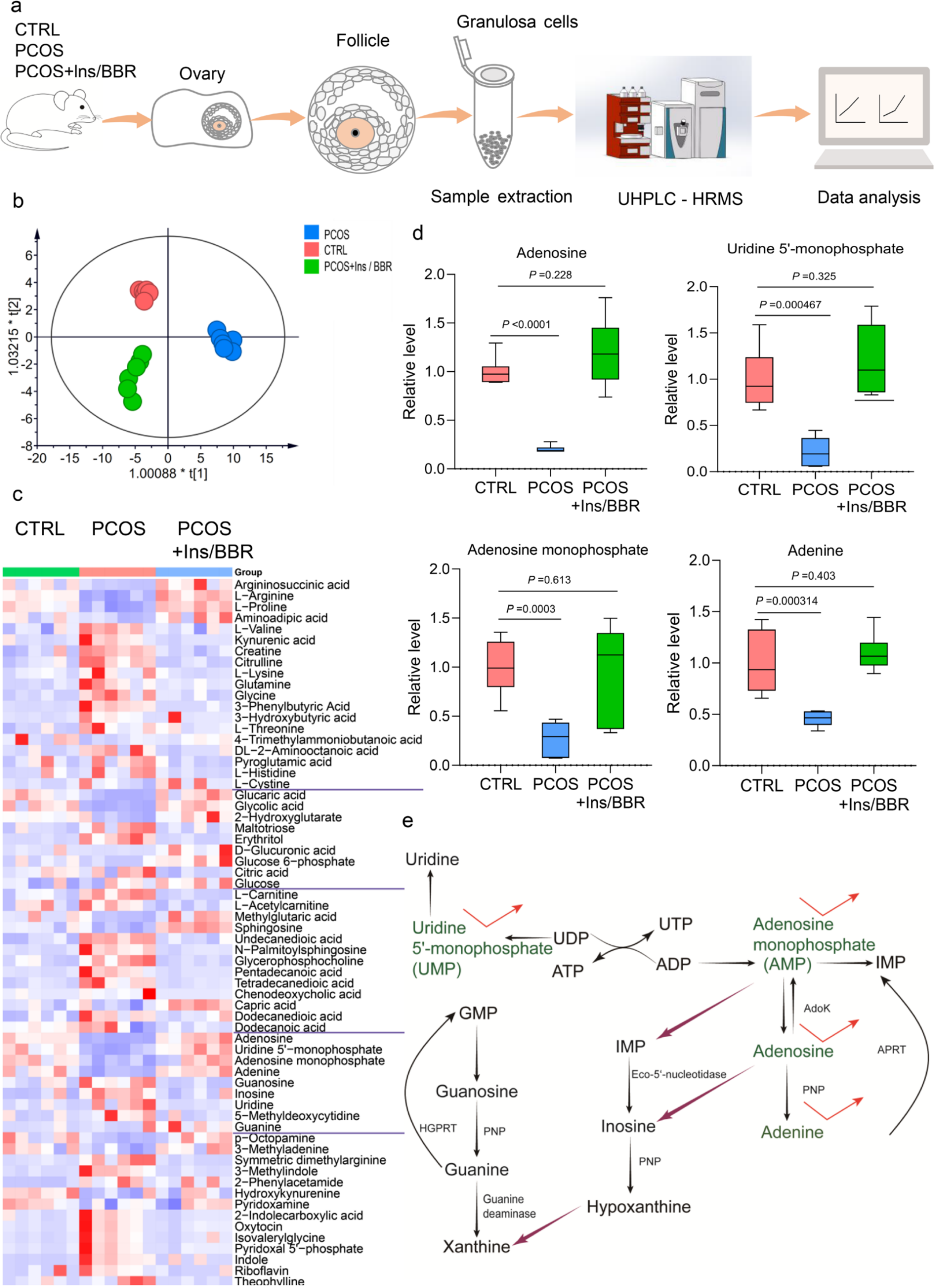
Metabolomic profiling in granulosa cells from PCOS mice. (a) Workflow for UHPLC–HRMS–based metabolomic analysis of granulosa cells collected from GV‐stage cumulus–oocyte complexes. (b) OPLS‐DA score plots showing separation between groups. (c) Heatmap of metabolite profiles in CTRL, PCOS and PCOS+Ins/BBR groups. (d) Relative levels of representative purine and pyrimidine metabolites (adenosine, adenosine monophosphate, adenine, uridine monophosphate) in CTRL, PCOS and PCOS+Ins/BBR groups. (e) Schematic diagram of purine metabolism derived from metabolomics data; altered metabolites are indicated with red dashed arrows. Data are mean ± SEM; Student's *t*‐test vs. CTRL. n.s., not significant.

Granulosa cells provide essential nutrients, energy substrates and paracrine signals that support oocyte growth and developmental competence. To assess whether Ins/BBR treatment modulates metabolic activity in PCOS granulosa cells, we performed extensive non‐targeted metabolomic profiling using UHPLC‐HRMS (Figure 6a; Suppl Table 1). OPLS‐DA analysis revealed clear separation among groups, with a cross‐validated predictive ability of Q^2^(cum) > 0.4, indicating robust metabolic differences (Figure 6b). Heatmap visualization further demonstrated characteristic metabolic reprogramming across treatments, including amino acid, fatty acid and nucleotide metabolism (Figure 6c).

#### 2.6.1 Effects of Ins/BBR on Nucleotide Metabolism in Granulosa Cells From PCOS Mice

Purine and pyrimidine nucleotides are essential for cellular energy homeostasis, nucleic acid synthesis and signal transduction. Compared with controls, granulosa cells from PCOS mice showed a pronounced reduction in metabolites involved in purine and pyrimidine metabolism, including adenosine, uridine 5′‐monophosphate, adenosine monophosphate and adenine (Figure 6d). This depletion suggests impaired nucleotide turnover and reduced energy availability. Strikingly, Ins/BBR supplementation largely restored these metabolites to near‐control levels, indicating normalization of nucleotide pools (Figure 6d).

#### 2.6.2 Effects of Ins/BBR on Fatty Acid/Amino Acid Metabolism in Granulosa Cells From PCOS Mice

**FIGURE 7:**
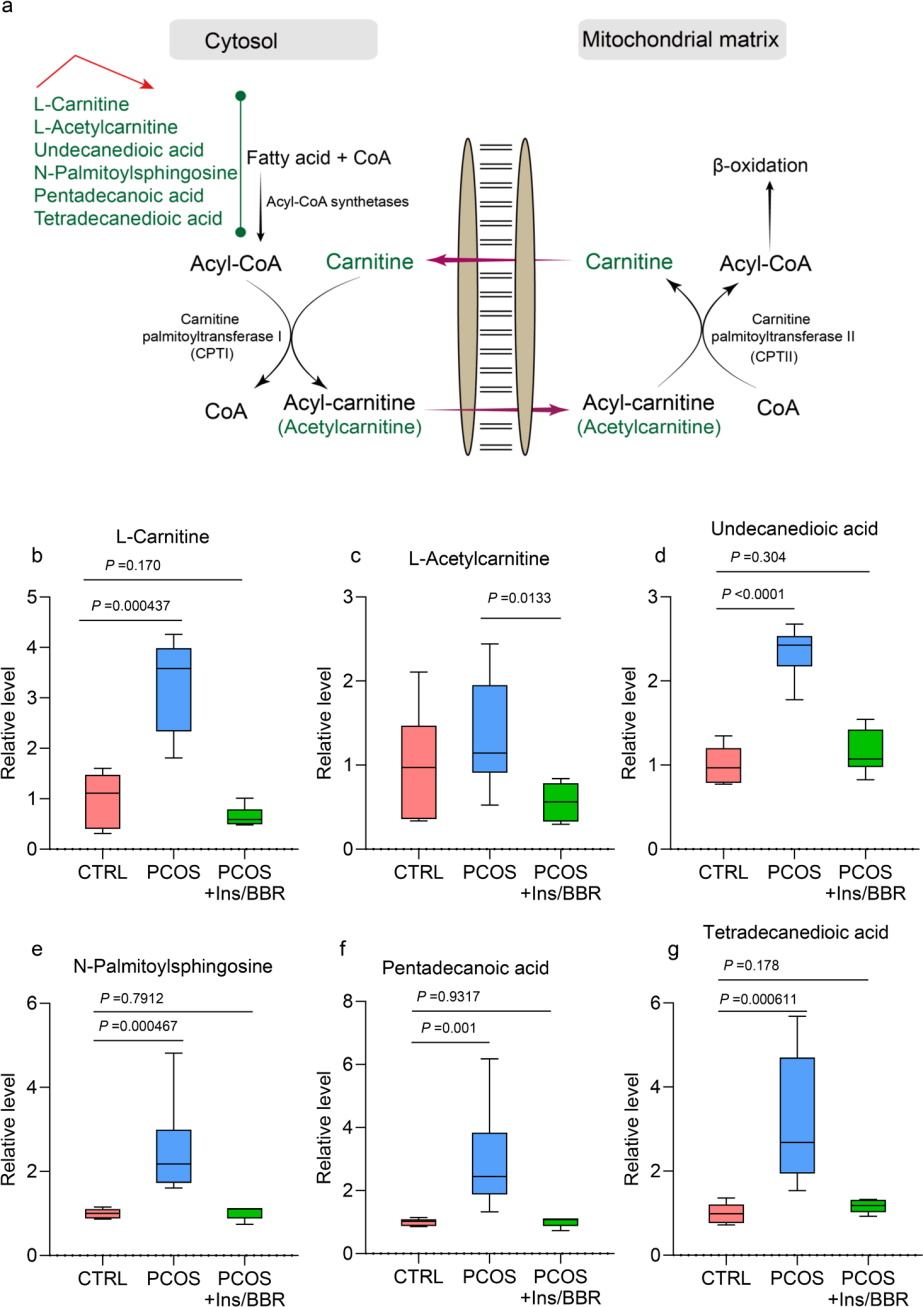
Altered fatty acid metabolism in granulosa cells from PCOS mice. (a) Schematic diagram of carnitine transport and fatty acid β‐oxidation in granulosa cells. Data are mean ± SEM; Student's *t*‐test vs. CTRL. (b–g) Relative levels of long‐chain fatty acids and carnitine‐related metabolites (L‐carnitine, acetylcarnitine, undecanedioic acid, pentadecanoic acid, tetradecanedioic acid and N‐palmitoylsphingosine) in CTRL, PCOS and PCOS+Ins/BBR groups.

Long‐chain fatty acids serve as a major energy substrate and require transport into mitochondria via the carnitine shuttle system for subsequent β‐oxidation (Figure 7a). Unlike nucleotide depletion, PCOS granulosa cells displayed a significant accumulation of long‐chain fatty acids, including undecanedioic acid, pentadecanoic acid and tetradecanedioic acid (Figure 7d,f,g). Consistently, levels of carnitine and acetylcarnitine, key molecules mediating fatty acid transfer during β‐oxidation, were markedly elevated, indicating disturbed fatty acid utilization (Figure 7b–c). Importantly, Ins/BBR supplementation significantly reduced these elevated metabolites (Figure 7b–g). In addition, Ins/BBR combination was also capable of restoring the metabolic patterns of amino acids in granulosa cells from PCOS mice. In specific, several metabolites in arginine/proline metabolism and urea cycle (argininosuccinic acid) were found to be decreased in PCOS granulosa cells and then markedly elevated following Ins/BBR treatment (Suppl Figure 1).

Altogether, these findings highlight that Ins/BBR exerts a broad metabolic reprogramming effect in PCOS granulosa cells, targeting energy production, lipid utilization, and amino acid homeostasis. Such improvements in granulosa cell metabolism may represent a key mechanism by which Ins/BBR enhances follicular function and oocyte competence in PCOS.

## 3 Discussion

MyoIns and DClns act through distinct biological mechanisms, and supplementation with these isoforms in a physiological ratio has demonstrated clinical benefits in PCOS management [31, 32, 33]. Berberine (BBR), a natural isoquinoline alkaloid, enhances insulin sensitivity and reduces hyperandrogenism [21]. Based on this rationale, we evaluated whether combining Ins and BBR produces additive or synergistic effects. Our data show that Ins/BBR more effectively corrected endocrine disturbances in PCOS mice, including elevated testosterone, LH, and LH/FSH ratio, along with reduced FSH and estradiol (E2). These improvements suggest that Ins and BBR act on complementary molecular targets to restore sex steroid homeostasis.

Beyond endocrine regulation, Ins/BBR treatment markedly improved ovarian morphology, characterized by reduced cystic follicles and normalized follicular development. The treatment also restored the thickness ratio of granulosa cells to theca cells, which is critical for folliculogenesis. Prior studies have established that steroidal and nonsteroidal factors secreted by granulosa cells and theca cells regulate follicular growth and differentiation across the basement membrane [34, 35, 36]. In theca cells, LH drives androgen synthesis, which provides substrates for estradiol biosynthesis by granulosa cells via aromatase (CYP19A1). This process is coordinated by steroidogenic acute regulatory protein (StAR), cytochrome P450 side‐chain cleavage enzyme (CYP11A1), and 3β‐hydroxysteroid dehydrogenase (3β‐HSD) [37, 38, 39]. Dysregulated expression of these enzymes, as observed in PCOS, impairs oestrogen production and follicle selection, leading to follicular arrest. In our study, PCOS mice displayed increased *StAR* and *3β‐HSD* expression with reduced CYP19A1, consistent with previous reports [40, 41, 42]. Notably, Ins/BBR normalized *StAR* and *3β‐HSD* levels and partially restored *Cyp19a1* expression, whereas single treatments showed weaker effects. These results underscore the advantage of Ins/BBR in rebalancing ovarian steroidogenesis.

Oocyte competence is tightly regulated by granulosa cells, which provide energy substrates via glycolysis and the tricarboxylic acid (TCA) cycle [43, 44]. Our metabolomic data revealed broad metabolic dysregulation in PCOS granulosa cells, including depletion of nucleotide pools, accumulation of long‐chain fatty acids, and reduced arginine/proline metabolism. Such alterations reflect impaired nucleotide synthesis, fatty acid β‐oxidation, and amino acid flux, which may compromise oocyte maturation and embryonic development. Importantly, Ins/BBR supplementation largely restored nucleotide levels, normalized fatty acid utilization, and corrected arginine/proline metabolism. Restoration of argininosuccinic acid may enhance polyamine synthesis, which is vital for cell proliferation and follicle growth. These findings suggest that Ins/BBR reprograms granulosa cell metabolism, and potentially, to support oocyte quality and reproductive outcomes.

Mitochondrial dysfunction represents another hallmark of PCOS. Mitochondria are central to energy supply, redox balance, and apoptotic signalling in oogenesis [45, 46, 47]. PCOS mice in our study exhibited pronounced mitochondrial abnormalities in granulosa cells, including disrupted cristae, reduced mtDNA copy number and decreased ATP production, consistent with clinical observations in women with PCOS and endometriosis [48]. Ins/BBR treatment alleviated these defects by restoring mitochondrial morphology, increasing mtDNA copy number, and elevating ATP levels. Such improvements in mitochondrial bioenergetics likely underpin the observed metabolic reprogramming and improved granulosa cell function. In addition, mitochondrial defects may be linked to lipid accumulation by limiting fatty acid β‐oxidation, but this possibility remains speculative in the absence of direct assessment of FAO‐related enzyme activity.

Collectively, our findings indicate that Ins/BBR targets multiple pathogenic pathways in PCOS: (1) restoring systemic sex hormone balance, (2) improving ovarian histology and follicle development, (3) normalizing steroidogenic enzyme expression, (4) reprogramming granulosa cells' metabolism and (5) correcting mitochondrial dysfunction. The integration of systemic endocrine regulation with local ovarian microenvironment remodelling may explain why Ins/BBR achieved superior efficacy compared with single‐agent treatments.

*Limitations and perspectives: Our* study was conducted in a mouse model, and extrapolation to human PCOS should be done cautiously. Future clinical studies are warranted to validate the translational potential of Ins/BBR and to optimize dosage and treatment regimens. Moreover, mechanistic studies linking specific metabolic pathways with oocyte developmental competence will further refine therapeutic strategies. Metformin is the standard insulin‐sensitizing therapy and a common benchmark in PCOS models [49]. Future studies should include direct comparisons with metformin to establish the relative efficacy of inositol‐ and berberine‐based interventions.

*Conclusion and clinical significance: In* summary, Ins/BBR therapy ameliorated endocrine, metabolic, and reproductive phenotypes in PCOS mice by synergistically regulating steroidogenesis, mitochondrial function, and granulosa cell metabolism. These findings provide preclinical evidence supporting the potential of Ins/BBR as a combinational intervention in PCOS. Given the high prevalence of polycystic ovary syndrome (PCOS) and its impact on fertility and long‐term health, we can further investigate the safety and efficacy of Ins/BBR combination therapy in clinical settings.

## 4 Materials and Methods

### 4.1 Animals

All animal experiments were conducted following the principles outlined in the Guide for the Care and Use of Laboratory Animals and were approved by the Institutional Animal Care and Use Committee at Nanjing Medical University. Female C57BL/6 (B6) mice, 3 weeks of age, were individually housed under a 12 h light/dark cycle at room temperature (20°C–23°C) with controlled humidity (20%–30%).

### 4.2 Generation of PCOS Mouse Model and Treatment

PCOS was induced by feeding a high‐fat diet (HFD, D12492i; Research Diets Inc.) combined with daily subcutaneous injections of dehydroepiandrosterone (DHEA; 25 μL, 36 mg dissolved in 0.5 mL DMSO and 0.5 mL PEG mixture) for 21 consecutive days, as previously described [50].

Mice were then randomly assigned to treatment groups:

*Ins group*: MyoIns+DClns (40:1; 310 mg/kg/day; 1800 mg MyoIns and 45 mg DClns dissolved in 45 mL saline).

*BBR group*: BBR‐loaded solid lipid nanoparticles (BBR‐SLNs, prepared by a solvent injection method using stearic acid as the lipid matrix and polyvinyl alcohol as the stabilizer, 150 mg/kg/day; 600 mg dissolved in 20 mL saline) [51, 52].

*Ins/BBR group*: MyoIns+DClns+BBR (40:1:18; 450 mg/kg/day; 1800 mg MyoIns, 45 mg DClns, and 810 mg BBR‐SLNs dissolved in 45 mL saline).

*NAC group: N‐acetylcysteine* (300 mg/kg/day; 1350 mg dissolved in 45 mL saline and 9 mL anhydrous ethanol).

All agents were administered by gavage needles for 28 consecutive days.

### 4.3 Serum Hormonal Analysis

Blood was collected after overnight fasting under anaesthesia with narcolan (0.1 mL/10 g body weight) via orbital vein bleeding. Serum levels of LH, FSH, testosterone (T), and estradiol (E2) were determined using ELISA kits (Nanjing Jiancheng Bioengineering Institute, China) following the manufacturer's protocols.

### 4.4 Granulosa Cells Collection

Superovulation was induced with intraperitoneal injection of 5 IU pregnant mare serum gonadotropin (PMSG). Cumulus–oocyte complexes were collected from antral follicles, and granulosa cells were mechanically separated by repeated pipetting.

### 4.5 Ovarian Histology

Ovarian tissues were fixed in 4% paraformaldehyde at 4°C overnight, dehydrated through ascending ethanol concentrations, cleared in xylene and embedded in paraffin. Five‐micrometre sections were then prepared, stained with haematoxylin and eosin (H&E) following standard methods [53], and cover slipped with neutral resin. Images were acquired using an Olympus IX71 fluorescence microscope.

### 4.6 Body Weight, Body Composition and Estrous Cycle

Body weight was recorded weekly for 4 weeks. Body composition was assessed using NMR (Biospec 7 T/20 USR, Bruker, Germany). Estrous cycles were monitored daily for 10 consecutive days by collecting vaginal smears with saline‐moistened swabs, followed by methanol fixation and eosin staining. Estrous cycle stages were determined by the relative composition of leukocytes, cornified epithelial cells and nucleated epithelial cells.

### 4.7 Glucose and Insulin Tolerance Tests

To perform the glucose tolerance test (GTT), mice were fasted for 16 h and subsequently injected with glucose (2 g/kg) intraperitoneally, and blood glucose levels were measured from tail vein at 0, 30, 60, 90 and 120 min (Accu‐CHEK Active, Roche). For the insulin tolerance test (ITT), mice fasted for 6 h received insulin (0.75 IU/kg, i.p.), and blood glucose was measured at the same time points. The area under the curve (AUC) was calculated. GTT and ITT were performed on the same mice with a 3‐day interval.

### 4.8 Transmission Electron Microscopy

Ovaries were prefixed in 2% glutaraldehyde for 8 h, postfixed in 1% osmium tetroxide for 1 h, dehydrated through graded ethanol, and embedded in epoxy resin. Ultrathin sections were examined under a transmission electron microscope (JEM‐1400plus, JEOL).

### 4.9 mtDNA Copy Number Quantification

Total DNA from granulosa cells was extracted using a DNeasy kit (QIAGEN). mtDNA content was quantified by real‐time PCR as described previously [53]. To generate the standard curve, PCR products using B6 forward and B6 reverse primers were connected into a T‐vector. Standard curve used five 10‐fold serial dilutions of purified plasmid standard DNA. Data are expressed as mtDNA/nuclear DNA, by measuring the threshold cycle ratio of a mitochondrial coding gene to a nuclear encoded gene.

### 4.10 ATP Measurement

ATP content in granulosa cells was determined using a bioluminescent assay kit (Sigma, MO, USA) according to the manufacturer's protocol [54]. Luminescence was detected at 450 nm, and ATP concentration was calculated from a standard curve.

### 4.11 Quantitative Real‐Time PCR

Total RNA was extracted from granulosa cells using the Arcturus PicoPure RNA isolation kit (Applied Biosystems) in accordance with the manufacturer's instructions. First‐strand cDNA was generated with a cDNA Synthesis Kit (Vazyme, China) and preserved at −20°C for subsequent analysis. For quantitative PCR, cDNA of *3β‐HSD*, *StAR* and *Cyp19a1* genes and *β‐actin* was mixed with SYBR Green (Vazyme, Q111) in a 20 μL reaction volume on an ABI QuantStudio 7 Flex PCR system (Applied Biosystems). Primer sequences were:

*3β‐HSD*: Fwd 5′‐TATTCTCGGTTGTACGGGCAA‐3′; Rev. 5′‐GTGCTACCTGTCAGTGTGACC‐3′; *StAR*: Fwd 5′‐ATGTTCCTCGCTACGTTCAAG‐3′; Rev. 5′‐CCCAGTGCTCTCCAGTTGAG‐3′; *Cyp19a1*: Fwd5′‐ATGTTCTTGGAAATGCTGAACCC‐3′; Rev5′‐AGGACCTGGTATTGAAGACGAG‐3′.

### 4.12 Metabolomics

After ultrasonic homogenization using a cell disruptor, the lysates were centrifuged at 16,000 × g for 15 min at 4°C to remove insoluble debris. Subsequently, 250 μL of the clarified supernatant was transferred into a new pre‐cooled 1.5 mL microcentrifuge tube. Metabolites extracted with 80% methanol were then vacuum‐dried using a centrifugal concentrator (Labconco, USA) and stored at −80°C prior to MS analysis. Samples were divided into three groups: Control, PCOS and PCOS+ Ins/BBR, three samples for each group.

Untargeted metabolomic analysis was conducted using an ultra‐high‐performance liquid chromatography system (UltiMate 3000, Dionex, Germany) coupled to a Q‐Exactive Orbitrap mass spectrometer (Thermo Fisher Scientific, Germany) for metabolite detection and quantification. Chromatographic separation was achieved on a Hypersil GOLD C18 column (100 mm × 2.1 mm, 1.9 μm; Thermo Fisher Scientific) operated at 40°C with gradient elution. The mobile phase consisted of solvent A (water containing 0.1% formic acid) and solvent B (acetonitrile containing 0.1% formic acid), delivered at a flow rate of 0.4 mL/min. The gradient program was set as follows: 99% A for 0–3 min, linearly decreased to 1% A by 10 min and held for 3 min (10–13 min), followed by re‐equilibration at 99% A for 2 min (13–15 min).

Mass spectrometric analysis was performed on the Thermo UHPLC‐Q Exactive system equipped with an electrospray ionization (ESI) source, and mass spectral data were acquired in dual‐polarity mode under full‐scan acquisition at a resolution of 70,000, with an m/z range of 70–1500. All samples were injected in a randomized order to minimize potential bias and systematic variation due to injection sequence and instrument stability.

Raw data acquired by the mass spectrometer were submitted to TraceFinder 3.1 Software. The metabolite identification was conducted by the comparison of accurate mass and retention time with the standard compounds. Statistical analyses were conducted using R software (version 2.15). Multivariate modelling was performed with SIMCA‐P (version 14.0; Umetrics AB, Sweden), including orthogonal partial least squares discriminant analysis (OPLS‐DA), to evaluate data reproducibility within groups and to characterize metabolic differences between groups. The variable importance in projection (VIP) value > 1.00 and P value < 0.2 with a 1.3‐fold change of each metabolite were used as the combined cutoffs of statistical significance. The corresponding KEGG (Kyoto Encyclopedia of Genes and Genomes) identifiers were subsequently uploaded to the KEGG Mapper tool to identify and reconstruct key biochemical pathways.

### 4.13 Statistical Analysis

Experiments were performed in triplicate or more, and data are presented as mean ± SD. Statistical comparisons were carried out using GraphPad Prism 8.0, employing either Student's *t*‐test or one‐way ANOVA where appropriate. *p < 0.05* was considered statistically significant; n.s., not significant.

## Author Contributions

J.G., C.Z., Y.Z. and H.Z. performed the experiments; C.Z., Y.Z., H.Z., T.T., G.Z. performed research; J.G, C.Z. and H.Z. analysed data, prepared figures and/or tables; J.G., Q.W. and H.W. conceived the project and wrote the manuscript.

## Funding

This work was supported by National Natural Science Foundation of China (82495190, 82374225, 82401945).

## Conflicts of Interest

The authors declare no conflicts of interest.

## Supporting information


**Figure S1:** Altered amino acid metabolism in granulosa cells from PCOS mice. (a–c) Relative levels of argininosuccinic acid, L‐proline and L‐arginine in CTRL, PCOS, and PCOS+Ins/BBR groups. (d) Schematic diagram of arginine and proline metabolism derived from metabolomics data; altered metabolites are indicated with red dashed arrows. Data are mean ± SEM; Student's *t*‐test vs. CTRL.

**Table S1:** Differential Metabolite Profiling

## Data Availability

The data that support the findings of this study are available from the corresponding author upon reasonable request.
