# Supplementary figures and images for: Inositol and Berberine Synergistically Reprogram Endocrine and Ovarian Metabolism in Polycystic Ovary Syndrome
Source: Cell Prolif. 2026 Feb 27;59(7):e70188. doi: 10.1111/cpr.70188 (PMC13325473; doi:10.1111/cpr.70188)

**Figure.S1**

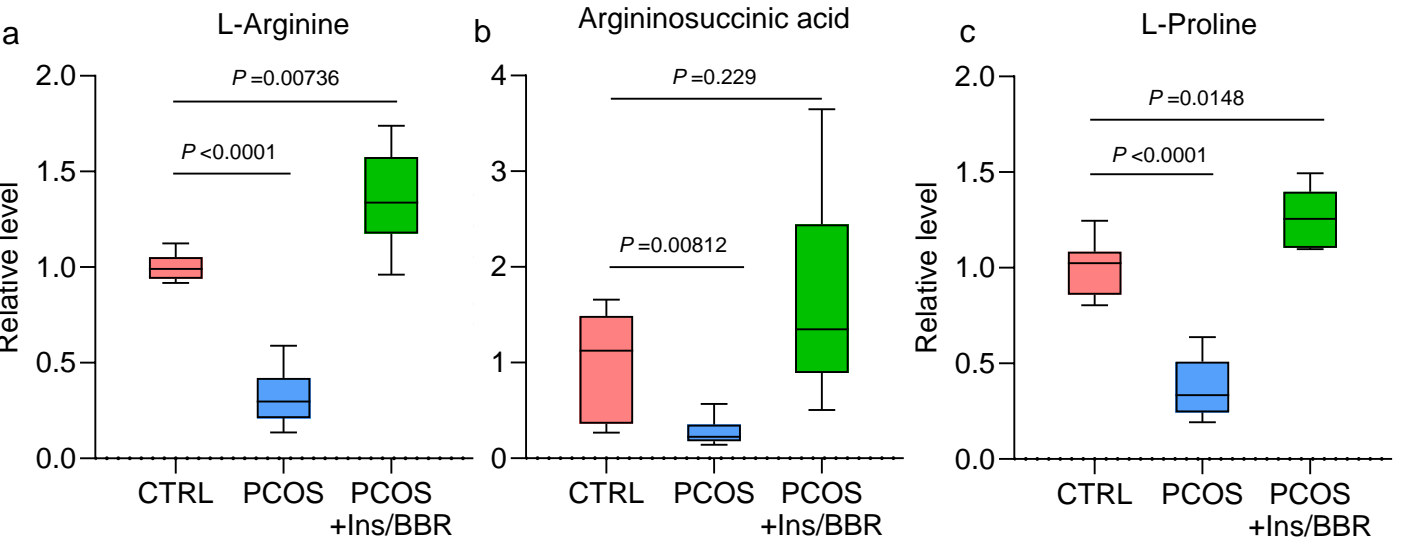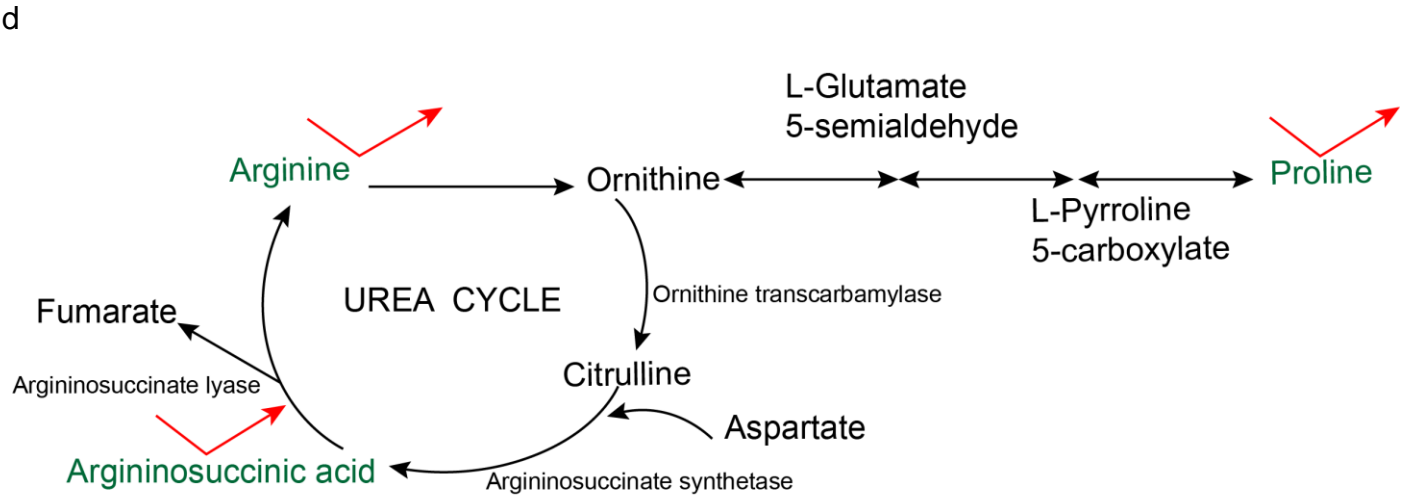

Supplement: Supplementary file 1 — Figure S1: Altered amino acid metabolism in granulosa cells from PCOS mice. (a–c) Relative levels of argininosuccinic acid, L‐proline and L‐arginine in CTRL, PCOS, and PCOS+Ins/BBR groups. (d) Schematic diagram of arginine and proline metabolism derived from metabolomics data; altered metabolites are indicated with red dashed arrows. Data are mean ± SEM; Student's t‐test vs. CTRL. [file CPR-59-e70188-s001.pdf]
